# Gram-scale synthesis of splat-shaped Ag–TiO_2_ nanocomposites for enhanced antimicrobial properties

**DOI:** 10.3762/bjnano.11.96

**Published:** 2020-07-29

**Authors:** Mohammad Jaber, Asim Mushtaq, Kebiao Zhang, Jindan Wu, Dandan Luo, Zihan Yi, M Zubair Iqbal, Xiangdong Kong

**Affiliations:** 1 School of Material Science and Engineering, Zhejiang Sci-Tech University, Hangzhou 310018, China; 2 College of Textile Science and Engineering, Zhejiang Sci-Tech University, Hangzhou 310018, China; 3 Zhejiang-Mauritius Joint Research Center for Biomaterials and Tissue Engineering, Hangzhou 310018, China

**Keywords:** antimicrobial properties, biomaterials, nanocomposites, silver nanoparticles, titanium dioxide

## Abstract

The control over contagious diseases caused by pathogenic organisms has become a serious health issue. The extensive usage of antibiotics has led to the development of multidrug-resistant bacterial strains. In this regard, metal-oxide-based antibacterial nanomaterials have received potential research interest due to the efficient prevention of microorganism growth. In this study, splat-shaped Ag–TiO_2_ nanocomposites (NCs) were synthesized on the gram scale and the enhanced antibacterial properties of TiO_2_ in the presence of silver were examined. The formation of Ag–TiO_2_ NCs was analyzed through various characterization techniques. The cell viability experimental results demonstrated that the Ag–TiO_2_ NCs have good biocompatibility. The antibacterial activity of the prepared Ag–TiO_2_ NCs was tested against the Gram-positive *Staphylococcus aureus* (*S. aureus*) and Gram-negative *Escherichia coli* (*E. coli*) bacterial strains. The Ag–TiO_2_ NCs exhibited promising and superior antibacterial properties compared to TiO_2_ nanospheres as confirmed by the bacterial growth and inhibition zone. The improvement in the antibacterial activity was attributed to the synergistic effect of the hybrid nature of TiO_2_ nanoparticles in the presence of Ag.

## Introduction

The rapid industrial development required to supply the necessities of the global population has certainly impacted the natural environment. The countries with a high population have been facing the risk of infectious diseases caused by pathogenic organisms. The existence of airborne pathogenic microorganisms with a high reproduction rate, such as virus, fungi and bacteria, has an influence on the human health and environment. However, it is a challenge to find remedies against these bacteria to control the permanent adhesive reaction. The Food and Drug Administration (FDA) has approved some potential antibacterial agents based on polymers and layer-by-layer coating to prevent pathogenic bacteria.

Antibacterial agents, such as antibiotics, quaternary ammonium compounds and metal ions have been widely used. However, the extensive use of antibiotics against some bacteria might increase the bacterial resistance to these antibiotics, leading to environmental toxicity and damage of target organs in the human body [1]. Also, these materials rapidly dissolve in the human body creating numerous clinical risks [2–3]. In spite of the desirable antibacterial properties, the traditional quaternary ammonium salts (QASs) and the antimicrobial peptides (AMPs) are highly toxic [4–5].

Recently, research in the field of nanotechnology has focused on trying to find solutions for some of the most serious environmental issues, such as energy conversion, and for the optimization of biomedical applications. Their small size, shape variability, surface functionalities, high surface-to-volume ratio and tunable physiochemical properties are unique characteristics that make nanomaterials promising for biomedical applications [6–7]. Multifunctional nanomaterials have been developed to decontaminate surfaces infested with infectious pathogens. Particularly, metal and metal-oxide-based disinfectants containing inorganic nanoparticles (NPs) have been broadly used since they simultaneously reduce the toxicity risk related to the organic materials and control the bacterial resistance against antibiotics [8]. Among the various types of nanomaterials, silver (Ag), zinc oxide (ZnO), copper oxide (CuO), iron oxide (Fe_3_O_4_) and titanium oxide (TiO_2_) are well recognized options due to their outstanding antibacterial properties. These nanoparticles have antibacterial activity due to the production of reactive oxygen species (ROS) [9–11]; more specifically, Ag NPs have been widely used in many fields, such as dental filling, wound dressing, water treatment and textile fabrics [12–13]. However, issues have been raised concerning Ag-associated genotoxicity and cytotoxicity in human cells [14]. To solve these toxicity problems, nanocomposite materials (NCs) have been considered as an alternative since they contain a small amount of Ag in a highly biocompatible material. Along these lines, TiO_2_ NPs have been used worldwide in biomedical applications due to their biocompatibility and cost-effectiveness [15]. Moreover, TiO_2_ NPs are inorganic materials which have significant antimicrobial activity against Gram-positive and Gram-negative bacteria [16–17]. Importantly, Ag and TiO_2_ NPs have been reported to be less toxic to humans [2,18–20]. In comparison to single NPs, the nanocomposites have advantages in terms of multifunctional use, antimicrobial and photocatalytic activities [3]. The main challenges of using the nanocomposites in the biomedical and textile-coating fields are to keep the synthesis processes at a low cost and to control for yield and stability issues. Currently, a number of techniques such as electron beam evaporation, magnetron sputtering, molecular precursor techniques and photo-deposition techniques have been applied to the preparation of nanocomposites [6,21–22]. However, these techniques are very sophisticated and not optimized for synthesis on a large scale.

Herein, a simple hydrothermal process was employed to obtain homogeneous Ag–TiO_2_ nanocomposites with a high yield. The prepared compounds were characterized by XRD, SEM, EDS, FTIR and UV–vis spectrophotometry. The cell viability upon exposure to the splat-shaped Ag–TiO_2_ nanocomposites was evaluated by using the Cell Counting Kit-8 assay. The antimicrobial activity of the as-prepared nanocomposites was investigated against Gram-positive *S. aureus* and Gram-negative *E. coli*.

## Experimental

### Synthesis of the Ag–TiO_2_ nanocomposite

A hydrothermal method was used to prepare the Ag–TiO_2_ nanocomposite on a gram-scale. 1.25 mol/L of a 16.0 mL titanium sulfate solution (Ti(SO_4_)_2_), 24.0 g of urea (CO(NH_2_)_2_) and 2.0 mL of 1.0 g/L polyvinylpyrrolidone (PVP) were added to a 32 mL glycol solution. The solution volume was adjusted to 100 mL and then AgNO_3_ was added at different molar ratios of Ag and TiO_2_ (1:1, 1:2, and 1:4, respectively). The prepared solution was kept under magnetic stirring for 2 h until all the reagents were completely dissolved. The reaction mixtures were transferred to a 100 mL polytetrafluoroethylene lining tube, placed in an autoclave and heated at 200 °C for 8 h. After the reaction was completed, the obtained material was washed three times with ethanol and centrifuged for further use. Pure TiO_2_ NPs were also prepared by using the above method without the addition of Ag as a precursor.

### Sample characterizations

The crystal structure of the samples was investigated through X-ray diffraction (XRD, ARL X'TRA, Thermo Techno, USA) using a Rigaku D/Max 2500 powder diffractometer with Cu Kα radiation (λ = 1.5406 Å). Scanning electron microscopy (SEM, JSM5610LV, JEOL, Japan) was used to observed the morphology of the Ag–TiO_2_ NPs. The sample components were examined by energy-dispersive X-ray spectroscopy. Fourier-transform infrared spectroscopy (FTIR, Nicolet IS50) was used to measure the infrared spectra. To detect the absorption profile of the prepared samples, the UV–vis spectroscopy technique was used.

### In vitro cytotoxicity

The Cell Counting Kit-8 (CCK-8) was purchased from Beyotime Biotechnology (Shanghai, China). Human colon adenocarcinoma CaCo-2 cells were used to investigate the cytotoxicity of the Ag–TiO_2_ nanocomposites using the CCK-8 assays. The NCs were dissolved in Dulbecco's Modified Eagle Medium (DMEM) at various concentrations (0, 8, 16, 32, 64 and 128 µg/mL). The cells were seeded at a density of 104 cells per well in 96-well plates containing DMEM medium supplemented with 10% FBS in a humidified atmosphere with 5% CO_2_ at 37 °C. The cells cultivated in the same plate without the nanocomposites were used as control. The concentration of the nanocomposite solutions was adjusted to 100 µL by adding DMEM. Next, the media containing the NCs were added to the wells and the cells were incubated for 24 and 48 h. A 10% CCK-8 assay solution was added to the media at the indicated times and incubated for an another one hour. Finally, the absorbance was measured using a microplate reader at a wavelength of 450 nm.

### Antibacterial test

Gram-positive *Staphylococcus aureus* (*S. aureus*) (ATCC 6538) and Gram-negative *Escherichia coli* (*E. coli*) (ATCC 8099) were obtained from the Shanghai Amoy Strain Biotechnology Co. (Shanghai, China). The disc diffusion method was applied to check the antibacterial activity of the prepared pure TiO_2_ NPs and Ag–TiO_2_ nanocomposite against the Gram-negative *E. coli* and Gram-positive *S. aureus* strains. The bacterial solution was prepared at approximately 1.0 × 10^8^ CFU/mL and diluted with a purified phosphate buffer solution. Pure cultures of the microorganisms were subcultured on a liquid broth agar solution. The antibacterial activity of the TiO_2_ and Ag–TiO_2_ NPs was examined by using the disk diffusion method. The sample solutions were prepared separately by using 0.005 g of the TiO_2_ NPs or Ag–TiO_2_ nanocomposites in 3 mL of purified water. Afterwards, 6 mm filter paper discs were soaked in the sample solutions, dried and applied on the culture plates. Finally, the plates were incubated at 37 °C for 24 h and the zone of inhibition was measured around the disc to estimate the antimicrobial activity [22].

## Results and Discussion

**Figure 1:**
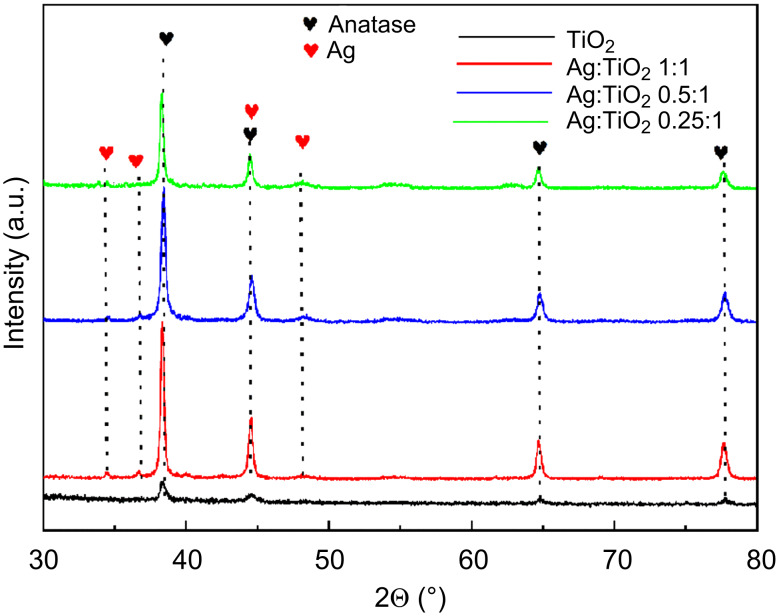
X-ray diffraction (XRD) patterns of pure TiO_2_ NPs and Ag–TiO_2_ nanocomposites at different ratios.

The XRD spectra were analyzed to verify the crystal structure and the phase purity of the prepared compounds. The XRD results of pure TiO_2_ and Ag–TiO_2_ nanocomposites are shown in Figure 1. The patterns showed characteristic peaks consistent with pure anatase TiO_2_ (JCPDS 21-1277) at 2θ values of 38.44(111), 44.74(200), 64.85(220) and 77.82(311).

The expected changes regarding a decrease in the crystallinity and the appearance of new peaks were observed when the Ag nanoparticles were added. The additional peaks corresponded to Ag at 2θ values of 36.78(103), which is perfectly indexed with the silver lattice planes (JCPDS 04-0783). Importantly, no impurity peaks were observed in addition to Ag and TiO_2_, indicating that the formation of the Ag–TiO_2_ nanocomposite was comparable to previously reported studies.

**Scheme 1:**
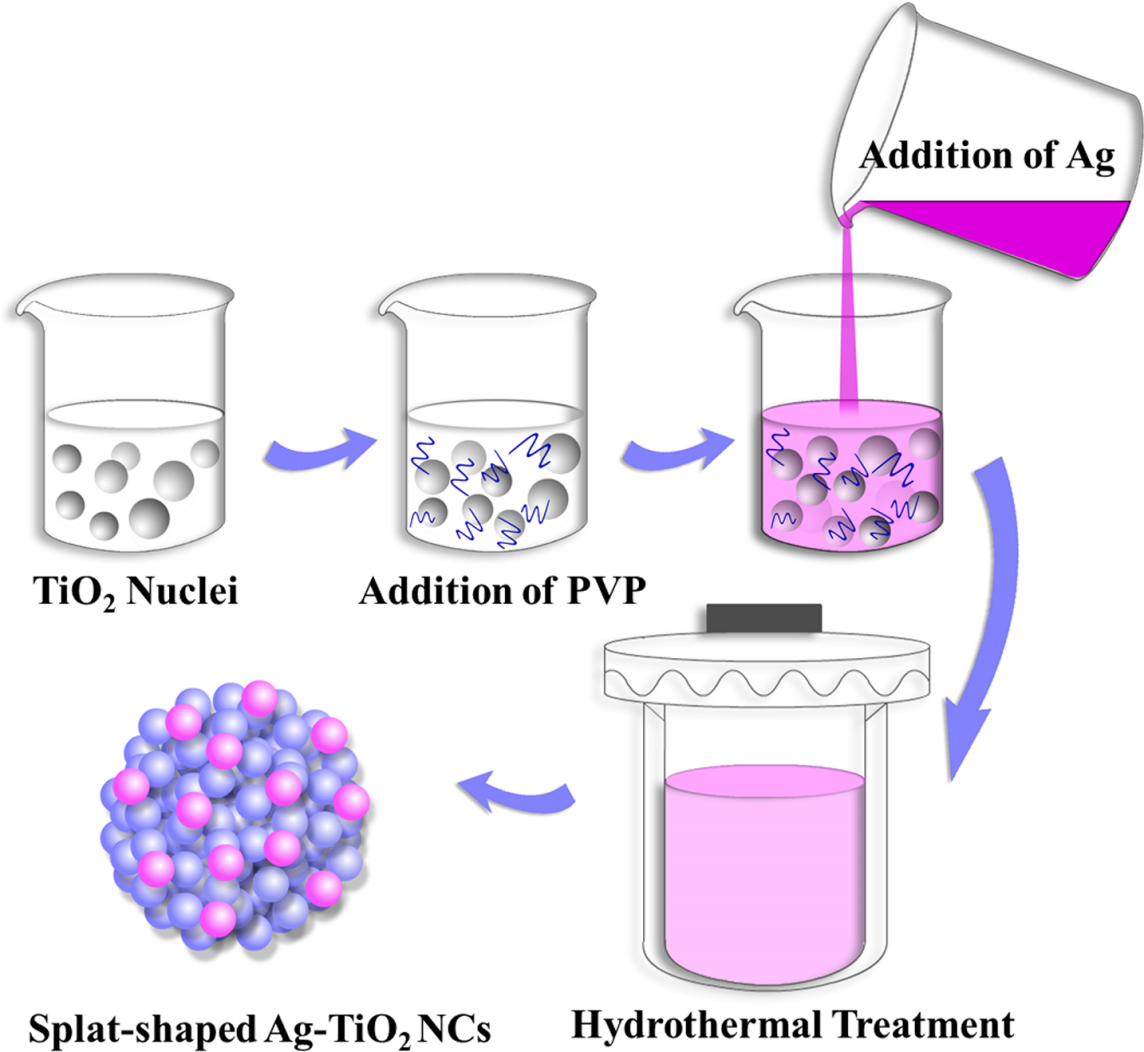
Synthesis of splat-shaped Ag–TiO_2_ nanocomposites prepared by the PVP-assisted hydrothermal method.

The synthesis of the TiO_2_ and Ag–TiO_2_ nanocomposites using the polyvinylpyrrolidone (PVP)-assisted hydrothermal method is illustrated in Scheme 1. In the growth process, the TiO_2_ NP nucleation started after adding the urea and PVP surfactant. Then, the aqueously dispersed AgNO_3_ solution was poured into the prepared TiO_2_/PVP mixture. It was assumed that the positively charged Ag^+^ ions interacted with the negatively charged PVP-modified TiO_2_ NPs due to electrostatic interactions. In addition, the PVP also acted as a reducing agent and facilitated the formation of small Ag nanoparticles at the surface of the TiO_2_ spheres to form a splat-shaped structure (Scheme 1). PVP played a significant role in generating these nanocomposites, serving as a growth modifier, stabilizer and reducing agent [23].

**Figure 2:**
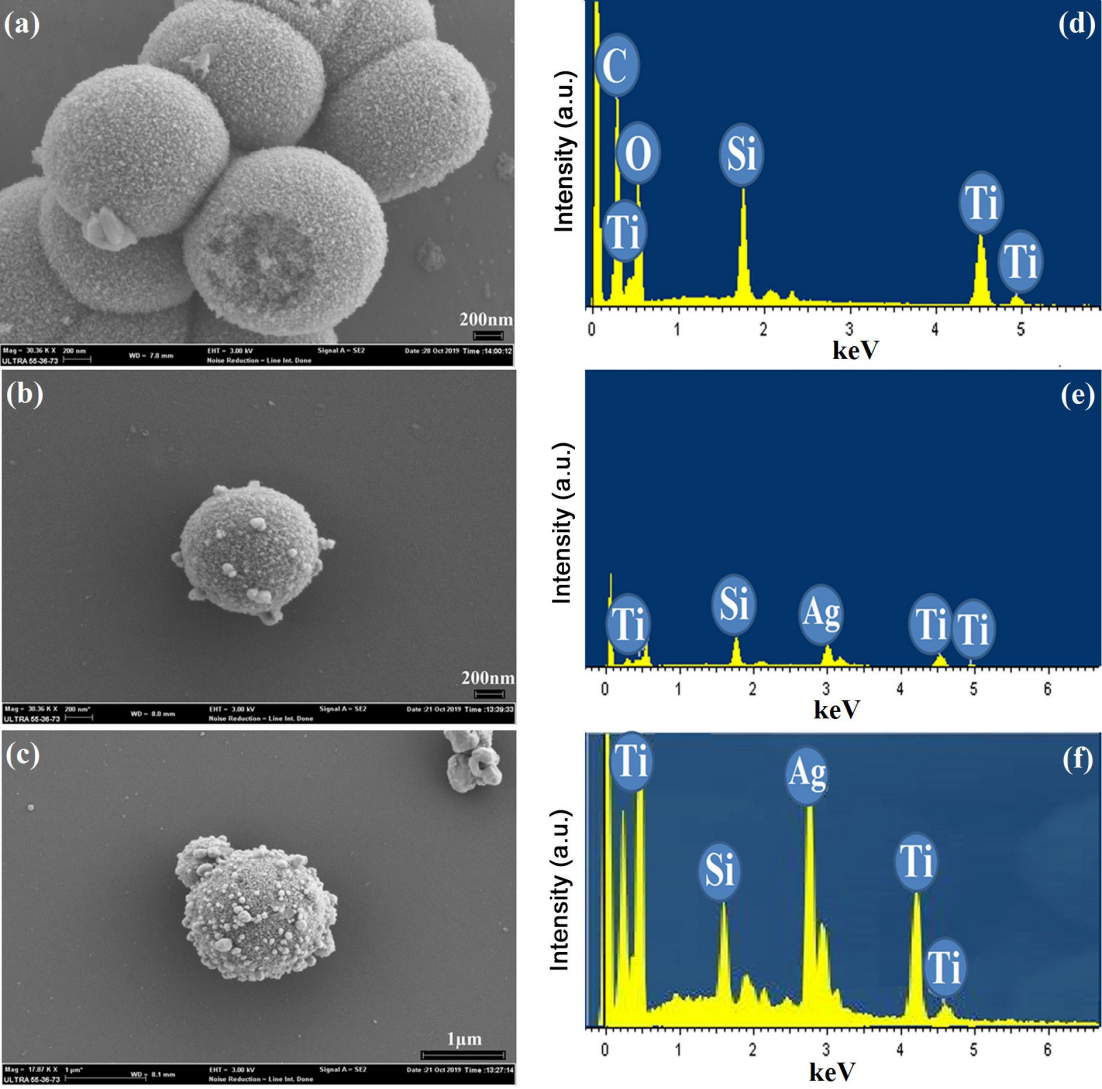
SEM and EDS images of the synthesized splat-shaped nanoparticles: (a) pure TiO_2_ NPs, (b, c) Ag–TiO_2_ nanocomposites with different Ag content. (d, e, f) are the respective EDS spectra. Scale bars are 200 nm (a, b) and 1 µm (c).

Figure 2 shows the morphology and the elemental analysis of the synthesized nanocomposites. The homogeneous and spherically shaped structure corresponds to the pure TiO_2_ NPs with an average diameter of 500 nm (Figure 2a). However, after the addition of Ag, small Ag nanoparticles (50 nm) appeared at the surface of theTiO_2_ nanospheres (Figure 2b,c). It was observed that the amount of the Ag NPs increased when the AgNO_3_ concentration increased. Figure 2d,f shows the EDS spectra. As expected, the EDS results identified Ag, Ti and O as the components present in the prepared compounds. The sample without Ag was identified as pure TiO_2_ nanospheres (Figure 2a).

**Figure 3:**
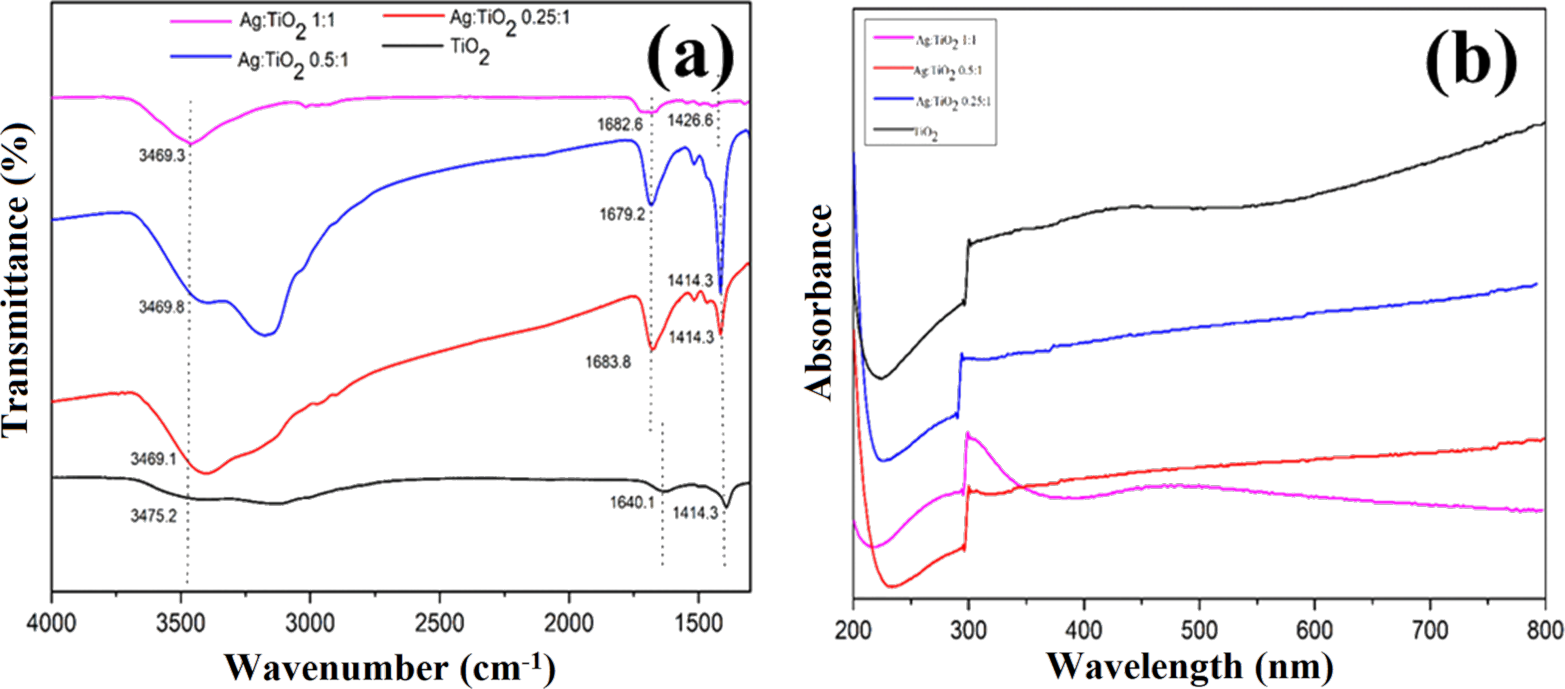
FTIR and UV–vis spectroscopy spectra of the pure TiO_2_ NPs and Ag–TiO_2_ nanocomposites.

Figure 3a shows the FTIR spectrum of pure TiO_2_ NPs in comparison with the Ag–TiO_2_ nanocomposites at different Ag concentrations. The central absorption peak of pure TiO_2_ NPs and Ag–TiO_2_ nanocomposites at 3475.2, 3469.1, 3469.8 and 3469.3 cm^−1^ showed a relatively similar behavior [24]. This result showed that the water molecules were adsorbed by the hydroxyl groups (OH) present at the surface of the analyzed samples. The peaks at 1640.1, 1683.8, 1679.2 and 1782.6 correspond to the bending vibrational peak of the O–H bonds [9]. The UV spectra of the Ag–TiO_2_ nanocomposite and pure TiO_2_ NPs shows the optical properties of these materials (Figure 3b). The absorption wavelength corresponding to pure TiO_2_ and Ag–TiO_2_ nanocomposites was ≈300 nm and this value was in accordance with the reported literature [7].

**Figure 4:**
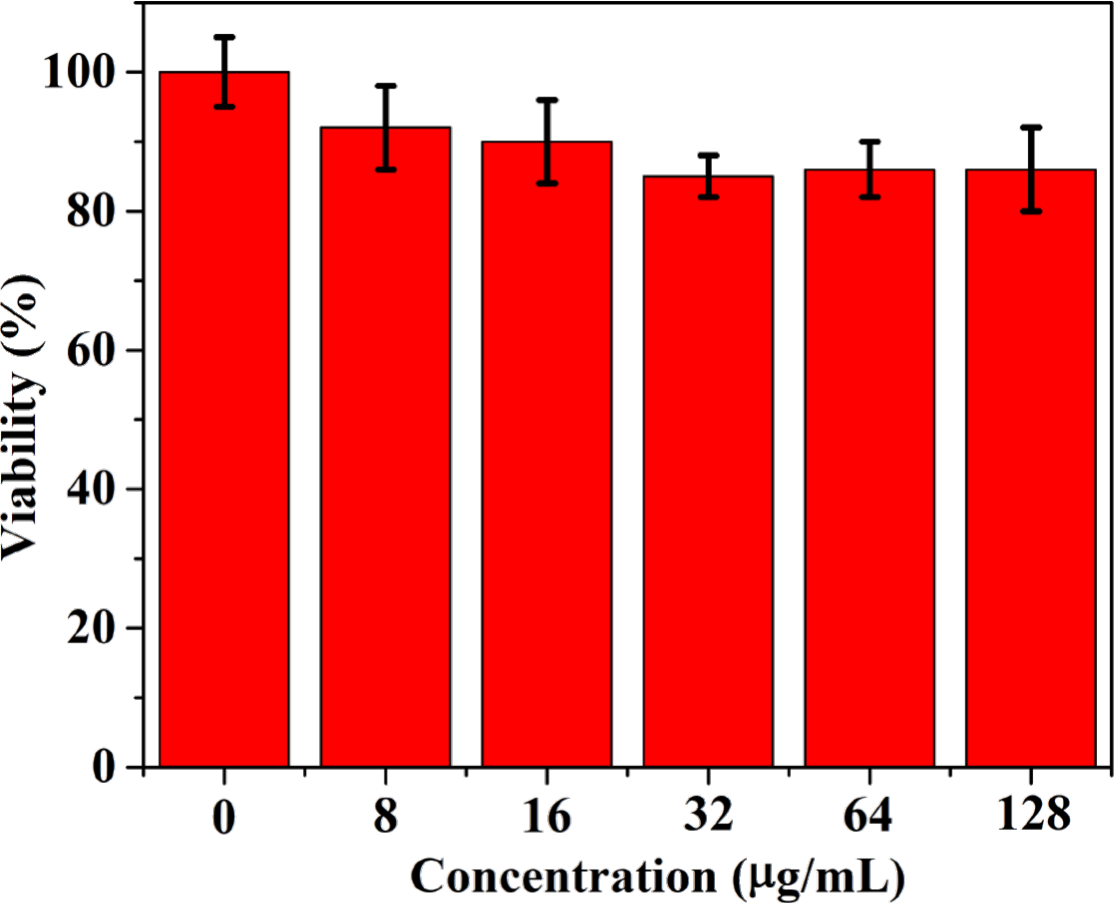
The in vitro cytotoxicity of the Ag–TiO_2_ nanocomposites at different concentrations incubated for 24 h with CaCo-2 cells.

The cytotoxicity of the NPs was investigated by using the CCK-8 assay after incubating the as-synthesized splat-shaped Ag–TiO_2_ NCs, at various concentrations, with human cells. Figure 4 shows the viability of CaCo-2 cells incubated with the NCs for 24 h. The results show that the viability of CaCo-2 cells incubated with NPs was higher than 80% even at high NP concentrations. The cytotoxicity results demonstrate that the NCs have a good biocompatibility which is needed for biomedical applications.

A number of intrinsic factors such as shape, size and surface chemistry of the nanostructures strongly influence the antibacterial activity. The aim of this study was to improve the antimicrobial activity of the biocompatible TiO_2_ material by growing the small Ag nanoparticles onto its surface. It is known that the Ag free radicals are used to kill bacteria due to their highly oxidative nature; however, when used at high concentrations, it can be toxic for the host cells.

**Figure 5:**
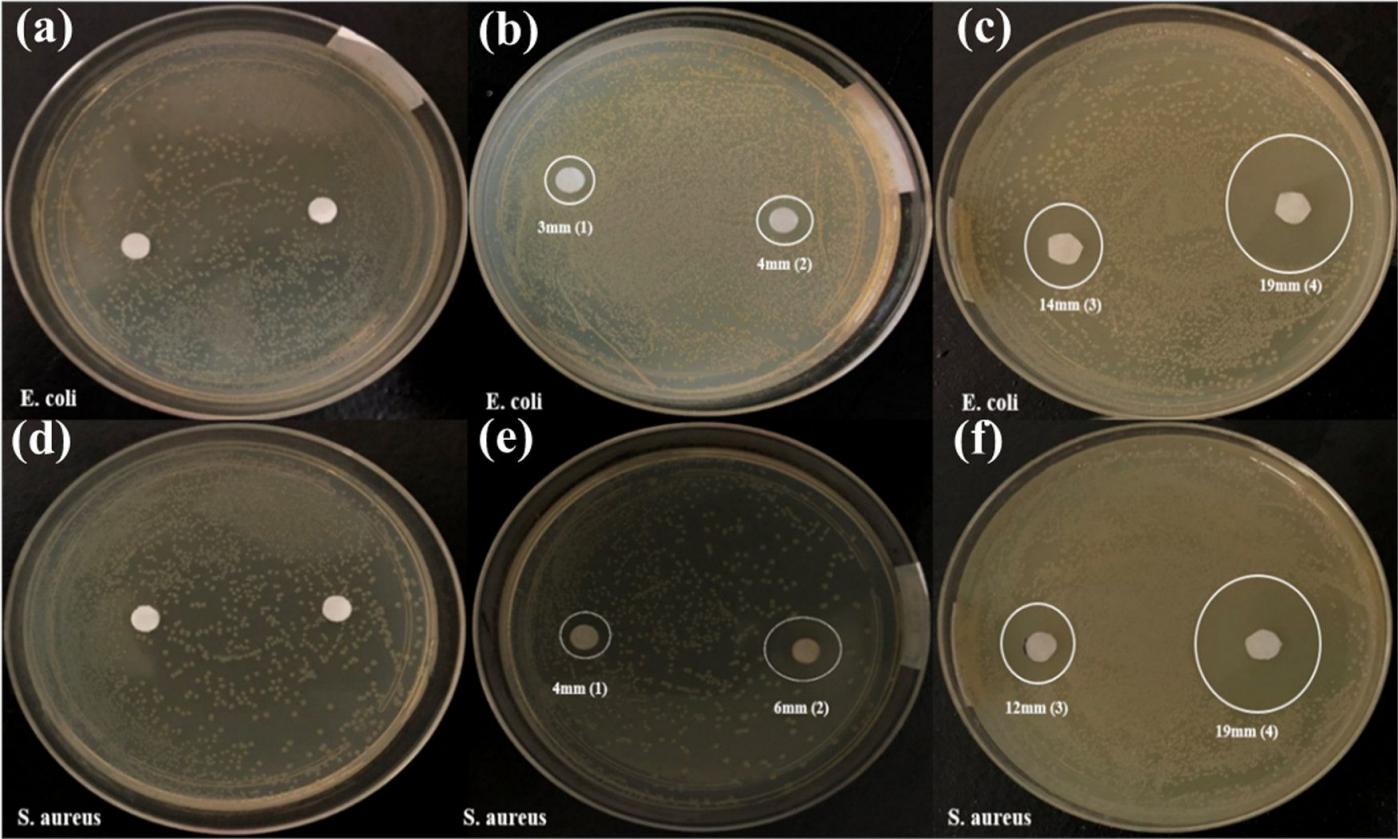
Antibacterial activity of the TiO_2_ and Ag–TiO_2_ nanocomposites against Gram-negative (a, b, c) and gram-positive (d, e, f) bacteria.

The antibacterial performance of the prepared and pure TiO_2_ was verified by measuring the progression of the bacterial inhibition zone. Table 1 and Figure 5 quantify the diameters of the inhibition zones for Gram-positive (*S. aureus*) and Gram-negative (*E. coli*) bacteria. Pure TiO_2_ nanoparticles and Ag–TiO_2_ nanocomposites showed clear inhibition zones of 3 mm, 4 mm, 14 mm and 19 mm against the Gram-negative *E. coli* bacteria (Figure 5b and c, respectively). In addition, 4 mm, 6 mm, 12 mm, and 19 mm inhibition zones were developed against the Gram-positive bacteria *S. aureus*. During the incubation with Ag–TiO_2_ nanocomposites, the silver ions were released from the NCs and gradually diffused out in the seeded agar. Then, the silver ions attached to the bacterial membrane, damaging the proteins and inactivating the bacteria metabolism, which also leads to the generation of reactive oxygen species (ROS) [25]. Hence, the above results showed that the antibacterial activity of the TiO_2_ is improved by the addition of Ag. The antibacterial results clearly demonstrated that the inhibition zone areas against both *E. coli* and *S. aureus* depend on the Ag concentration in the Ag–TiO_2_ nanocomposites. These results proved that nanocomposites have a better antibacterial performance than the single NPs.

**Table 1:** Inhibition zones against Gram-positive and Gram-negative bacteria.

Prepared samples	Gram-positive (*S. aureus*) inhibition zone (mm)	Gram-negative (*E. coli*) inhibition zone (mm)

sample 1 (TiO_2_)	3	4
sample 2 (Ag/TiO_2_ 1:4)	4	6
sample 3 (Ag/TiO_2_ 1:2)	13	14
sample 4 (Ag/TiO_2_ 1:1)	19	19

## Conclusion

In this work we have designed and synthesized splat-shaped Ag–TiO_2_ nanocomposites (NCs) by using a simple hydrothermal method with different concentrations of Ag. Characterization techniques such as XRD, SEM, EDS and FTIR corroborated the expected results and showed that the Ag–TiO_2_ NCs exhibit good biocompatibility. The amount of small Ag metal particles at the surface of the TiO_2_ nanospheres and Ag NPs increased when the Ag concentration was increased. Finally, these Ag–TiO_2_ NCs were successfully tested in terms of their antimicrobial properties and the results demonstrated that the inhibition zone against both *E. coli* and *S. aureus* strongly depended on the Ag concentration and on the synergistic effect of the NCs. These types of nanocomposites may also have potential to be used in wound healing, photocatalytic and toxic dye removal applications.
